# A Case Study in Arts-Informed Ethics Education in the Nuclear and Radiological Sciences

**DOI:** 10.1007/s11948-025-00558-9

**Published:** 2025-11-13

**Authors:** Nicole E. Martinez, Sarah E. Donaher, Jonathan S. Nagata, Lindsay Shuller-Nickles

**Affiliations:** 1 Department of Environmental Engineering and Earth Sciences, Clemson University, Clemson, SC USA; 2 Department of Civil and Environmental Engineering, University of Tennessee, Knoxville, TN USA; 3 Department of Environmental Health and Engineering, Johns Hopkins University, Baltimore, MD USA

**Keywords:** Nuclear sciences, Radiation protection, Ethics, Education

## Abstract

There is a need for cross-disciplinary researchers and professionals in the radiological sciences who can navigate complex interconnected ethical-social-technical issues, communicate across a wide audience in consideration of multiple stakeholder perspectives, and remain self-critical, aware, and reflective of the field with the intent of continuous improvement within the broader profession. Given that traditional curriculum related to the nuclear and radiological sciences emphasizes the technological, scientific aspects of radioactivity and ionizing radiation, a graduate-level course in “nuclear culture” was developed that employs various forms of art, expression, and material culture as a vehicle for encouraging deeper, dedicated reflection on related social and ethical issues. This paper provides a description of course structure and representative content, along with discussion of student perceptions, as a case study in the use of an arts-informed approach (that is, a STEAM-based approach) to guiding students through perspective-taking, self-expression, and authentic and critical evaluation of broad issues surrounding current and historical use of radiation. The course consists of discussions, reflective writing, and projects, supplemented with hands-on activities. Student perceptions of the course, elucidated through thematic analysis of post-hoc surveys, revolved around: novel educational approaches; student engagement and validation; ethics, morals, and empathy; and the societal impact of nuclear. Review of student and instructor perceptions suggests that art in various forms can be incorporated into graduate-level curriculum to improve the educational experience of nuclear-focused students and promote deeper reflection and understanding of social and ethical issues related to their chosen field.

## Introduction

### Motivation

Ionizing radiation has been ubiquitous in popular culture since its discovery, capturing the imaginations of scientists, artists, and the public, at times a hero and at times a villain. In the early 1900s, young women were poisoned by radium, the same radionuclide used then (and now) to treat cancer (Martinez et al., 2022; Rowland, 1994). Nuclear fission, an arguably clean and reliable source of energy, was first harnessed into a weapon of mass destruction in the mid-1900s. Ionizing radiation has a power invisible to the naked eye, both fascinating and at times alarming, with an important, nuanced role in society.

In traditional curriculum related to the nuclear and radiological sciences, emphasis is placed on the technological, scientific aspects of radioactivity and ionizing radiation. Although strong science and engineering is obviously essential, reliance solely on the “hard sciences” has limits (ICRP, 2018; Oughton, 2013). There is a need for cross-disciplinary researchers and professionals in the radiological sciences who can navigate complex interconnected ethical-social-technical issues, communicate across a wide audience in consideration of multiple stakeholder perspectives, and remain self-critical, aware, and reflective of the field with the intent of continuous improvement within the broader profession (Bryant et al., 2024; ICRP, 2006, 2018; Wieder et al., 2022). Although the importance of teaching social and ethical responsibility to engineers and scientists is overwhelmingly acknowledged by universities, employers, and professional societies, there is a recognized need for more robust incorporation into current educational schemes (Kreth et al., 2024; Zandvoort et al., 2013). Additionally, with ongoing discussion of the decline of disruptiveness of modern science and technology (Park et al., 2023), reflecting on art and cultivating creativity have been suggested as paths to more transformative science and insightful scientists (Malone, 2013, 2022; Martinez, 2020; Yanai & Lercher, 2023).

To that end, a graduate-level course in “nuclear culture” was developed that employs various forms of art, expression, and material culture [physical objects with societal meaning, Dant (2015); Rentetzi and Ito (2021)] as a vehicle for encouraging deeper, dedicated reflection on social and ethical issues surrounding the nuclear and radiological sciences. Not initially intended as a formalized intervention for research purposes, post-hoc student surveys were used to gauge student perception as a preliminary evaluation of the approach to the course. This paper provides a description of course structure and representative content, along with discussion of student and instructor perceptions, as a case study in the use of an arts-informed approach (that is, an integrative science, technology, engineering, arts, and mathematics [STEAM]-based approach) to guiding students through perspective-taking, self-expression, and authentic and critical evaluation of broad issues surrounding current and historical use of radiation.

### Background

Although STEM and STEAM education are not consistently defined in the literature (Aguilera & Ortiz-Revilla, 2021; Mejias et al., 2021), traditional graduate-level curriculum within (technical) nuclear and radiological sciences integrates science, technology, engineering, and mathematics in applied approaches to problem solving, consistent with at least one school of thought with respect to STEM education (Aguilera et al., 2021). Such “nuclear and radiological sciences” broadly include, for example: nuclear engineering; radioactive waste management; health physics (i.e., radiation protection); medical physics; radiation ecology, biology, and chemistry; radiation detection and measurement; risk assessment; computational dosimetry; etc.

Social sciences, arts, and humanities are clearly not new to the consideration of nuclear (Carpenter, 2016; Perko et al., 2019; Verma, 2021), nor is ethics education for scientists and engineers a novel concept (Børsen et al., 2021). However, less prevalent is an integrated approach to the education of scientists and engineers with respect to the arts (i.e., STEAM education). STEAM education has been shown to positively impact critical thinking and creativity as well as student recruitment, sense of belonging, and the equitability of learning environments, elegantly reviewed elsewhere (Danielson et al., 2022; Mejias et al., 2021; Perales and Aróstegui, 2024). STEAM education often employs art as a tool for teaching or emphasizing scientific concepts or developing related skills, such as visualization and problem solving (Kuo, 2024; Sanz-Camarero et al., 2023; Trout et al., 2022; Wong et al., 2023; Zhang & Jia, 2024), or in some cases for evaluating or modifying student perceptions of engineering (Cojan et al., 2024; Matthew et al., 2020). Less common is using art to explore ethical aspects of, for example, the practice, application, and societal impact of science and engineering (Marcone, 2022). An arts-informed or arts-integrated approach to socio-ethics education has been explored in some disciplines, with positive outcome (Danielson et al., 2022; Delany & Gaunt, 2018; Kinsella & Bidinosti, 2016). To the authors’ knowledge, a nuclear-focused application has not been explicitly considered, although the relevance and usefulness of art has been recognized within the field (Malone, 2013, 2022; Martinez, 2020). Kinsella and Bidinosti (2016) describe arts-informed approaches to education as being linked, among other things, to “self-awareness,” “deeper levels of reflection,” and “development of empathy,” which are aspirational outcomes of the course discussed herein. For example, the importance of empowering members of the public and incorporating broader socioeconomic issues when assessing and managing radiological risk is well-acknowledged, but to do so effectively, reasonably, and ethically is an on-going challenge (ICRP, 2007, 2020; Smith & Martinez, 2017; Wieder et al., 2022). Fostering trust and developing empathy are considered two key components of this (among others), but practical strategies for how to incorporate these aspects into traditional nuclear and radiological sciences curricula are quite limited (Ando, 2018; Wieder et al., 2022; Zölzer & Zölzer, 2022). 

Case studies in education research “analyze a particular set of issues within the educational context” to potentially “serve as the basis of a pedagogical tool” (Grauer, 2012). Such case studies provide “real-life context” to which theory can be applied or further developed and should draw on “multiple lines of evidence” in forming conclusions (Merriam, 1985; Yin, 2018). Of note is that Hamilton and Corbett-Whittier (2013) provide a robust, plain language review of the background and use of case studies in education research with practical examples. The reflective case study described herein is largely exploratory, with descriptive elements, intended to provide initial insight into the efficacy of the implemented STEAM-based approach on student outcomes—particularly with respect to perspective-taking and ethics—through consideration of student and instructor perceptions.

## Methods

The phrase “nuclear culture” has been used in different contexts (Hughes, 2012; Ross, 2024). Here, the use is not meant to be philosophically precise, rather simply to refer to how radioactivity and nuclear energy are used, expressed, and/or perceived in the public eye. *Nuclear Culture* is an upper-level graduate course within the Department of Environmental Engineering and Earth Sciences intended primarily for engineers and scientists with nuclear or radiological expertise; the course assumes a baseline level of knowledge related to, for example, ionizing radiation interactions and effects, the nuclear fuel cycle, galactic cosmic radiation, the Manhattan Project, etc. As such the course has as a prerequisite of prior coursework that includes nuclear or radiological sciences in the scope. The course seeks to cultivate, beyond technical understanding, a greater appreciation for and broader perspective of the impact of radioactivity and ionizing radiation on the world and in particular to facilitate recognition and articulation of social and ethical dimensions underlying certain issues or situations. This includes the intention that students will, “through imaginative and compassionate engagement with the stories of diverse others, learn to adopt a moral attitude” (D’Olimio & Peterson, 2018).

A survey was distributed to past students of the *Nuclear Culture* course to elucidate their perceptions. Students who had previously completed the *Nuclear Culture* course were invited to complete a survey (Qualtrics, Provo, UT) comprised of one demographic question, five Likert-scale statements (ranging from *strongly disagree* as 1 to *strongly agree* as 5) and three open-ended questions. The survey was conducted outside of standard, end-of-semester course evaluations, and was conducted in accordance with Clemson University Institutional Review Board (IRB) exempt protocol number IRB2025-0355.

Descriptive statistics were used to examine the Likert scale (quantitative) results of the survey; a thematic analysis (Braun and Clarke, 2006; Kiger and Varpio, 2020) was conducted to examine open-ended (qualitative) survey responses utilizing an inductive coding approach. Initial line-by-line coding was conducted to develop a coding frame. A second round of focused coding was conducted to synthesize and clarify themes emerging from the data. Codes and data segments were sorted, iteratively, to arrive at cohesive thematic categories. Qualitative survey data was analyzed by an independent evaluator (JN) as a form of investigator triangulation to reduce bias; data and codes were managed with Dedoose software (Dedoose, 2025). Survey results (Sect. Findings) suggest that the course did indeed expose students to new topics and concepts—utilizing, for some, novel pedagogical approaches—that challenged them to think outside their comfort zone, recognize differing worldviews, grapple with ethical and moral dilemmas, and critically examine the broader impacts of nuclear technology on culture and society.

## Course Overview

### Learning Outcomes and Student Deliverables

Similar to Danielson et al. (2022), this course seeks “synergies between scientific concepts and arts modalities and practices” and to allow “students’ lived experiences to be recognized and validated.” Upon completion of the course, it is intended that students will be able to:

- Describe, analyze, and interpret different representations of radioactivity or nuclear energy, and situate these representations in relevant societal and historical contexts.
- Demonstrate technical competency by critically evaluating accuracy of various works, while also discussing the value of balancing realism and aesthetics/entertainment.
- Reflect on the cultural and personal impact of how radiation and nuclear energy are represented in different works of art.
- Produce a creative work that demonstrates a thoughtful reflection of what was learned in the class.

Outcomes are assessed through engagement in group discussion, regular written reflections, and three projects: a group project devised by the students, the leading of a discussion on a work of the students’ choosing (outside of those on the syllabus), and the production and presentation of an original creative work. Highly structured rubrics are not employed, rather evaluations are based on individual student progress, preparation, and respectful and engaged interaction with their colleagues. Similarly, student artefacts are not evaluated from an artistic perspective; evaluation is based on the thought and attention given to the activity and a demonstration of critical reflection on topics explored during the semester. The final creative work is not intended nor expected to be at a professional level.

Class meetings are dedicated to group discussions, engaging with supplemental materials (e.g., music, short videos, objects like jewelry or toys) and creative activities, ranging from short, simple “warm-up” exercises (color page or simple composition exercise) to more involved (cyanotype printing, canvas painting, construction with Legos, paper folding, playing themed board games), fitting the theme of the current course topic. This approach is consistent with the principles of active learning, i.e., “activities that students do to construct knowledge and understanding” (Brame, 2019) and object-based learning, i.e., “active integration of objects into the learning environment” (Chatterjee et al., 2015). Although the application of active learning has surpassed the research of its efficacy in some areas (Hartikainen et al., 2019), the benefits are generally considered to be improved learner participation and retention, opportunities for collaborative work, and increased student perception of the course material as having real world value (Howell, 2021; Michael, 2006). Object-based learning “encourages the interchange of personal reflections, values, beliefs, and ideas,” including across different cultures (Chatterjee et al., 2015).

Additionally, reflection is rarely taught or incorporated into traditional scientific or technical courses, making the inclusion of written student reflections a novelty of the course described herein. Intentional written reflections have been shown to promote metacognition in students, additionally improving student critical thinking and writing habits (Dyment & O’Connell, 2010), encouraging the consideration of course material from the perspective of benefit to self and society (Ryan, 2013), and shifting students from “consumers” to “producers” of knowledge (Costa & Kallick, 2008).

### Representative Course Content

The works considered in the course are based on a combination of availability and extent of published professional analysis, interviews, and/or curation (Brougher, 2013; Carpenter, 2016; Decamous, 2018; Jolivette, 2012; Volkmar, 2022), student interest, and instructor preference. What follows are representative, non-exhaustive selections to serve as example course content as well as to provide additional context for student perceptions that follow. Many of these examples are rooted in or draw from the narrative arts because of the importance and impact of stories:

…narrative artworks allow us both to engage with the thoughts, feelings and goals of key protagonists and to use our imaginative capacities to develop the sympathy and empathy for others –including others who are different form ourselves—which compassion requires. (D’Olimio & Peterson, 2018)

#### HBO’s Chernobyl

The HBO miniseries *Chernobyl* is a dramatization of the events surrounding the world’s worst nuclear accident. *Chernobyl* provides extensive material for discussions surrounding transparency, professional integrity, and human dignity, while also eliciting “feeling” or identifying with the characters. In other words, it provides an avenue for developing and discussing empathy. Ample opportunities exist for discussion of competing values and value judgements, the importance of considering other perspectives, and issues underlying the spread of disinformation and misinformation.

Points of consideration for students including both “real time” and “deeper” reflective thinking questions. Real time questions are intended to assist students with active notetaking and with recognizing and articulating emotional responses. For example, reflecting on the impact of color, sound/music, and cinematography, noting real-time reactions or perspective shifts, considering differences in how an expert and non-expert might react or feel and why, etc. One student indicated in their survey (see Sect. Findings) that they “really liked that the instructor gave specifics to look for as we went through material. Specifically I remember things like asking about how lighting / colors conveyed what was happening in an episode of Chernobyl.” Additional questions, intended to elicit deeper thinking include:

- The series opens with a character reflecting on the “cost of lies,” a phrase also on the series cover art. In general, what do you think the “cost of lies” is? What about in this specific instance? For those who may be unfamiliar with the events or outcomes of the Chernobyl disaster, what kind of feeling or emotion do you think this opening intends to convey?
- Early in the series a minor character says: “This is why no one likes scientists.” Which of the characters, if any, do you find likable thus far? Do you think this is as the creators intended? How important do you think “likability” is as a scientist? Why?
- What testimony should the lead scientist provide? It is easy to say ‘the right thing’ without regard for personal consequences, so take the time to think through this decision and reflect on the potential outcomes or consequences of his choice. What would you do in a situation like this? Although dramatized, this type of scenario (e.g., lack of academic freedom or punishment for whistleblowing) is not unrealistic, given politically motivated attacks on scientists and engineers in recent years.
- Of the many relationships presented, those between the three main characters strengthen as the series goes on, as they develop genuine trust and reliance on each other. With this as a backdrop, what role do you think our professional relationships play in (1) the successful practice of our science and (2) our individual well-being? What should our “professional network” look like? How do we choose (or do we get to choose?) who we work with?
- The final scene of Chernobyl is a sweeping view of the area with narration: “…this at last is the gift of Chernobyl. Where I once would fear the cost of truth, now I only ask, what is the cost of lies?” The piece of music that follows, playing over the epilogue, motivated the name of the episode: “Vichnaya Pamyat” which is Ukrainian for “Memory Eternal”. Show creator Craig Mazin said in an interview that this choice was intended as a memorial and an expression of empathy with the people of the region. Thinking back over the series, how would you now express “the cost of lies”? How does it compare to “the cost of the truth”? Has your perspective changed since the beginning of the series? How are these ideas relevant to the current practice of nuclear and radiological safety?

**Fig. 1:**
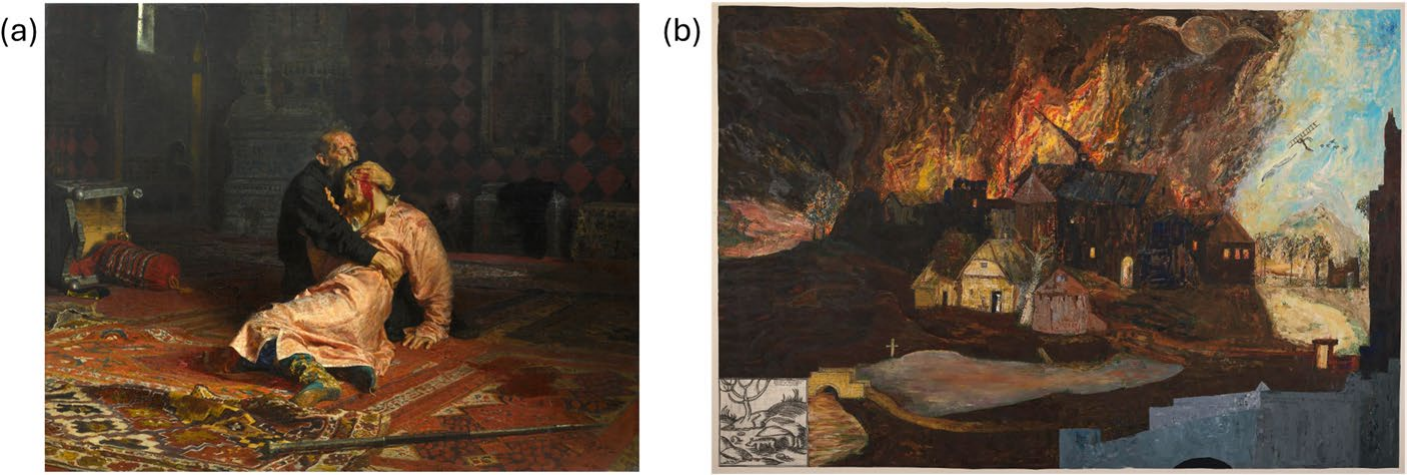
(**a**) Ilya Repin, Ivan the Terrible and His Son Ivan on 16 November 1581, 1883-85, oil on canvas. (**b**) William T. Wiley, Grebeny - The Burning Village after Bosch, 1994, acrylic, charcoal, and graphite on canvas, Smithsonian American Art Museum, Gift of Patrick C. Duffy in memory of Wally Goodman © 1994, William T. Wiley (with permission)

There are also opportunities to discuss and compare representations in the miniseries to oral histories, such as that compiled by Svetlana Alexievich in *Voices from Chernobyl* (Aleksievich & Gessen, 2005) as well as to link to other bodies of artistic work. For example, Episode 3 lingers on the painting *Ivan the Terrible and His Son Ivan* (Fig. 1a) following a discussion of sacrifice (originally highlighted by a student). To reflect on the symbolism or impression intended to be conveyed by the director, we read about and discuss the context of the painting and how it was used. As part of this discussion we also introduce a piece in a similar color palette by William T. Wiley; Wiley was an American artist who painted *Grebeny - The Burning Village after Bosch* in the mid-1990s (Fig. 1b) after hearing of the experience of those living in contaminated areas, drawing on the work of classic Hieronymous Bosch (Callard, 2009). A representative question for reflection includes:

- What stands out to you about Wiley’s piece? Although a very different medium than *Chernobyl*, do you see any similarities? What feelings (if any) does it evoke, and would you consider any of those feelings to fall under “empathy”?

#### Coppelion

**Fig. 2:**
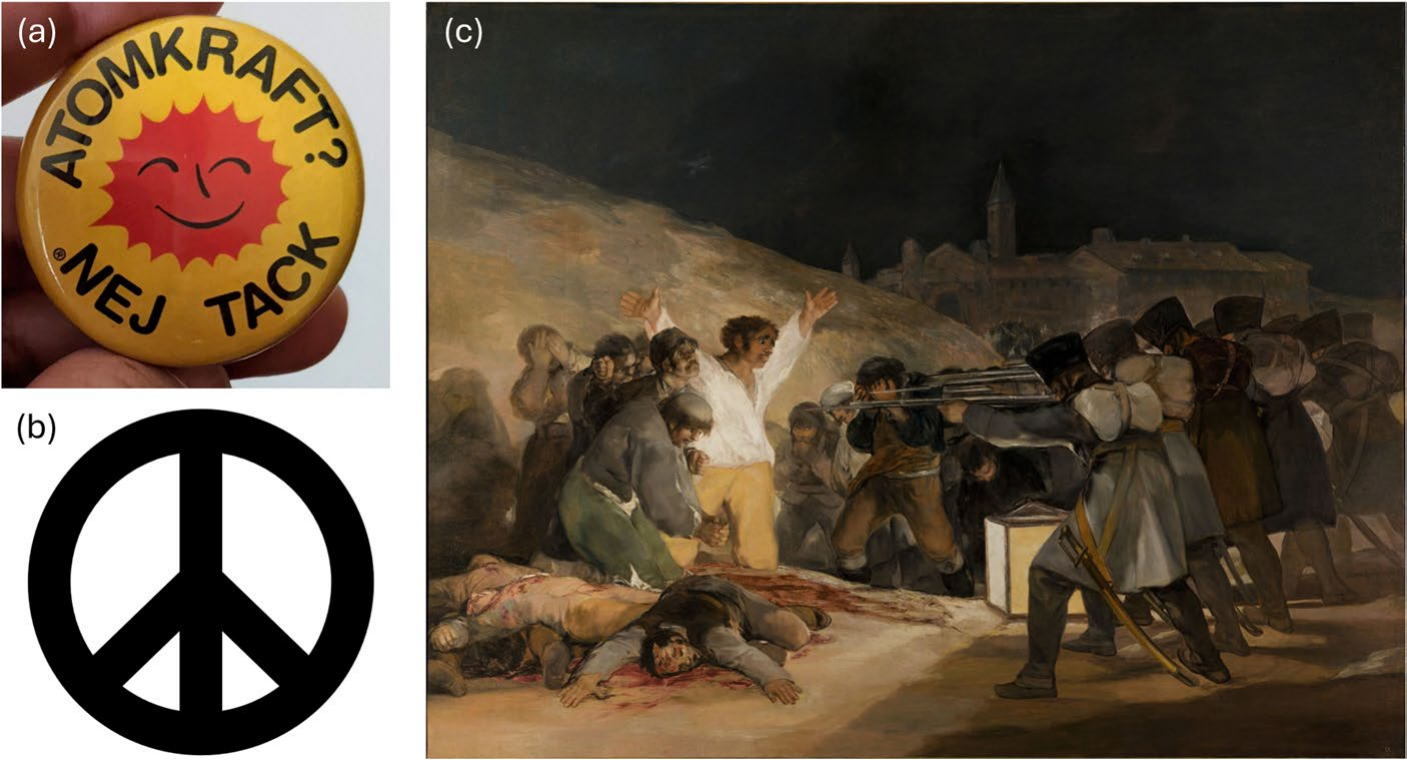
(**a**) Pin depicting the Smiling Sun symbol (“Atomic Power? No Thanks” in Swedish); (**b**) the Campaign for Nuclear Disarmament (CND) symbol (i.e., peace symbol); (**c**) Francisco de Goya y Lucientes, The 3rd of May 1808 in Madrid, or “The Executions”, 1814, oil on canvas

Coppelion (コッペリオン) is a science fiction manga written from 2008 to 2016 by Japanese author Tomonori Inoue. Due to a radiological disaster, Tokyo has become inhabitable except for certain genetically engineered teenagers (the three main characters are high school girls named Ibara, Aoi, and Taeko) who are deployed as a search-and-rescue team. The manga was adapted into a 13-episode anime, originally planned to air in 2011.^1^ The release was delayed due to the accident at Fukushima Dai-ichi, ultimately airing in 2013. Episode 4 (“Sunset”) of the anime is assigned to introduce discussion of environmental justice in the context of radioactive waste management as well as discussion of bioethics and autonomy, comparing the science fiction “biorobots” of the anime with the real-life conscripted soldiers at Chernobyl. In this episode, the protagonists discover that the company Yellow Cake (named after yellowcake uranium), which is responsible for handling the world’s radioactive waste, has been clandestinely and illegally dumping the waste in Tokyo (Fig. 2a). The emotional reaction from Ibara is strong, she expresses both sadness and anger with an intensity that is not characteristic of her thus far in the series. Discussion questions include:

- Did you watch *Coppelion* in English or Japanese (or both)? What role do you think language plays in the overall level of impact of TV shows and movies?
- Although given a different name, we are introduced to a new type of “biorobot,” similar to that of the liquidators in Chernobyl. Although science fiction, human genetic modification isn’t too far of a stretch to imagine (Cyranoski, 2019). What are some similarities and differences in these two sets of “biorobots,” including in how they are treated by those around them? What do you think are some of the broader ethical issues the author of *Coppelion* raises in his depiction of the Coppelions?
- Why is Ibara (one of the main characters) so upset? Isn’t this area already “trashed”?

The Yellow Cake company logo^2^ is featured only briefly as a non-essential part of the story, but discussion of this logo, e.g., aesthetic and intent, leads into discussion of other thematically similar logos and symbols such as the Smiling Sun symbol (Fig. 2a) and the Campaign for Nuclear Disarmament (CND) symbol, more commonly known as the “peace symbol,” (Fig. 2b) along with Goya’s *The Third of May* (Fig. 2c) (as partial inspiration for the CND symbol; Rigby, 1998) which can subsequently transition into discussion of war and nuclear weapons.

#### Nuclear Enchantment

**Fig. 3:**
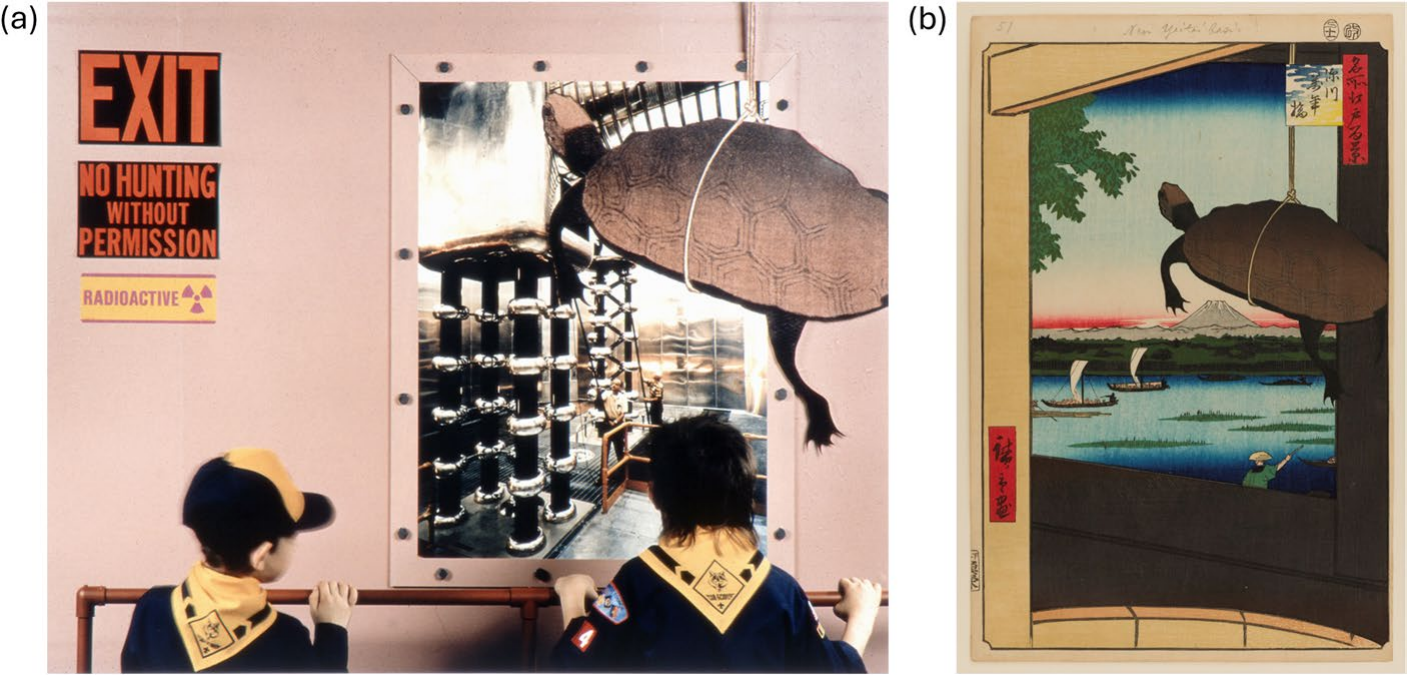
(**a**) Plate 13: “Hojo-e/Releasing of Life,” H Injector, LAMPF Accelerator, Clinton P. Anderson Meson Physics Facility, Los Alamos National Laboratory, New Mexico, Chromogenic print (Ilfocolor Deluxe), Patrick Nagatani ©1991 Estate of Patrick Nagatani / Artists Rights Society (ARS), New York. (**b**) Utagawa Hiroshige (Japanese, 1797–1858). Mannen Bridge, Fukagawa (深川萬年橋, Fukagawa Mannenbashi), No. 56 from One Hundred Famous Views of Edo, 11th month of 1857. Woodblock print. Brooklyn Museum, Gift of Anna Ferris, 30.1478.56 (Photo: Brooklyn Museum)

*Nuclear Enchantment* (Nagatani & Parry, 1991) is a series^3^ of photocollages by Japanese-American artist Patrick Nagatani, who was also a photography professor at the University of New Mexico (Roberts, 2017). With a family connection to Hiroshima, he often juxtaposed Native American and Japanese cultures in his art related to the nuclear legacy of the United States as he believed these two populations were those most significantly impacted by Manhattan Project and Cold War activities. In several of his collages (including Fig. 3a) Nagatani draws inspiration from the classic *One Hundred Famous Views of Edo* (名所江戸百景), a series of scenic, mica-embellished woodblock prints of what is now Tokyo, by Japanese artist Hiroshige (Martinez, 2020; Nagatani & Parry, 1991; Trede et al., 2015).

In Fig. 3a, the artist’s son and his friend are visiting the Los Alamos Meson Physics Facility (LAMPF) injector complex at Los Alamos National Laboratory. Workers are seen through a window, envisioned by the artist as parallels to the fishermen of Hiroshige’s No. 56 (Fig. 3b). The title of the piece (“Hojo-e,” or “Rite for the Release of Living Beings”) refers to a widespread Buddhist practice in which small, captive animals such as birds, turtles, or fish are released into fields and streams. This is intended to signify compassion and awareness of the interrelationship of all sentient beings (Law, 1994). Hiroshige’s No. 56 is reflective of this practice; additionally, the turtle and bridge (the “Bridge of Ten Thousand Years”) symbolize longevity (Andō et al., 2010).

The reference to No. 56 in “Hojo-e” returns to Nagatani’s common themes in *Nuclear Enchantment* of reverence for life, intergenerational and environmental justice, and the legacy of the nuclear weapons complex. *Nuclear Enchantment* is typically discussed in the same module with *Coppelion*; additional discussion questions include:

- What role does culture and/or life experience play in our perception of nuclear energy, and of radiation in general?
- Both *Nuclear Enchantment* and *Coppelion* contain negative depictions of the nuclear fuel cycle (or we might instead say that these works express concerns related to the fuel cycle). Some of these concerns are not unfounded, even if they make us uncomfortable. What are some examples of these concerns, and how do we feel about them? How can we (or do we?) reconcile a person or a community’s negative experience and genuine concerns about nuclear energy with our own personal experience and perspective in a way that is respectful and empathetic?
- Each artist’s cultural heritage is evident in their work (particularly in *Nuclear Enchantment*). Do you think, for either body of work, that the impact is different for those with different cultures and experiences? How so? What, and how, can we learn about cultures that are different than our own through this art?

#### How to Make a Bomb

*How to Make a Bomb* is an intricate “durational gardening project” from artist Gabrielle Hirst and curator Warren Harper “examining the structural connections between horticulture, state power, and nuclear colonialism.”^4^ This modern project is part of an effort to save a nearly extinct strain of rose species, *Rosa floribunda ‘*Atom Bomb’ which was initially bred and named during the overlapping rose breeding and atomic crazes of the mid-19th century (Hirst & Harper, 2019, 2021). One component of the project consists of a presentation by Hirst explaining the role of nuclear and horticultural colonialism in Britain’s history, which is followed by an instructional rose grafting workshop (The Old Waterworks, 2023). Hirst’s presentation itself is artful, deftly weaving together multiple scientific fields (e.g., botany, nuclear physics) and historical events (e.g., similar cultures of radical secrecy among rose breeders and scientists working on The Manhattan Project, the outsourcing of both British convicts and nuclear weapons testing to Australia, and principles of Aboriginal land stewardship) with her critique on British colonialism and imperialism. During the subsequent interactive propagation workshop, participants are also encouraged to engage with and further the piece by grafting their own *Rosa floribunda* ‘Atom Bomb’ plant material in various places around Britain (Hirst & Harper, 2019).

The work was continued with a multimodal two-part work entitled *An English Garden*, consisting of a garden installation in Gunners Park, Essex and a published book (Hirst & Harper, 2021). The physical installation primarily consists of flower beds of *‘*Atom Bomb’ roses alongside benches with plaques detailing the history of the location as a shipping point for nuclear weapons on their way for testing in Australia. The book includes watercolor botanical drawings, garden maps, and a pamphlet (Hirst & Harper, 2019) detailing the rose grafting information previously taught in the *How to Make a Bomb* workshop. Taken together, Hirst’s work serves multiple purposes: conservation of a rare strain of rose, awareness to the historical nuclear misdeeds inflicted upon Indigenous peoples, and information provision on present British policy of nuclear armament proliferation.

*How to Make a Bomb* is contained within the course’s “Nuclear Weapons” module highlighting the history, consequences, and ethical issues associated with nuclear weapons development, testing, and military use. Discussion questions include:

- There have been extensive philosophical discussions, academic and otherwise, concerning the ethics of both the decision to pursue the development of nuclear weapons as well as the decision to use them. Did any of the works herein affect your perception of the use/testing of nuclear weapons? Under what conditions, if any, do you think the use of nuclear weapons is appropriate? Why?
- Some of the selected works encourage a reflection on our (communal) past or legacy, particularly related to mistakes made. Was there anything you watched/read that gave you pause to think more deeply about something? If so, which piece(s) and what was that “something”? What do you think “reflection” means in the context of science and engineering and what role does or should it play in the scientific or engineering process?

#### Crystal Palace

**Fig. 4:**
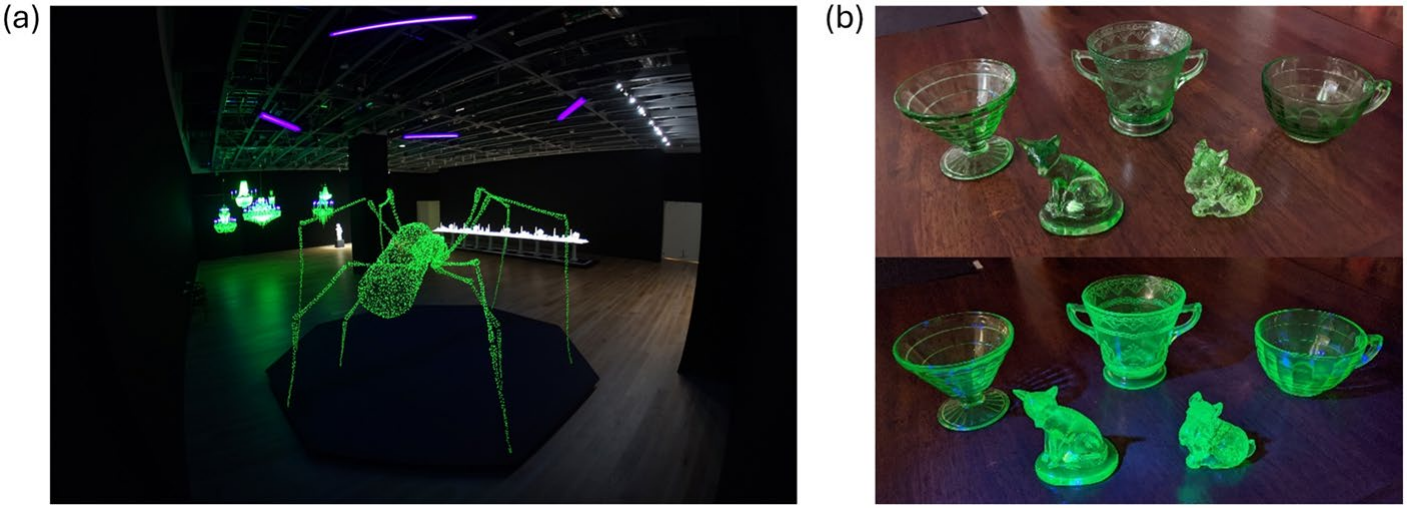
(**a**) *Spider Tale* (center) with *Crystal Palace* (left of center) in the background. Photo by Jinno Shingo. Image courtesy of Ken + Julia Yonetani. (**b**) Uranium glass (top) under UV light (bottom) (author’s collection)

Ken + Julia Yonetani^5^ developed a series of installations that consist of sculptures built with uranium glass and housed with ultraviolet (UV) lights intended to express the artists’ concern with the nuclear fuel cycle from an environmental justice perspective. These works include *Crystal Palace: The Great Exhibition of the Works of Industry of all Nuclear Nations*,* Spider Tale*,* What the Birds Knew*,* Wishes*, and *Electric Dreams.* Uranium glass fluoresces a neon green under UV light, starkly contrasting with the violet color of commercial UV bulbs (Fig. 4). Complementing this discussion is physical interaction with uranium glass objects, such as jewelry, figurines, and decorative glassware (Fig. 4b).

*Crystal Palace* consists of 31 chandeliers refitted with uranium glass beads and UV bulbs, representing countries with nuclear power; the size of the chandelier reflects the amount of nuclear power in the respective country (Carpenter, 2016). Developed in response to the nuclear accident at Fukushima-Daiichi, it is intended to inspire reflection on consumerism, technology, and the potential consequences thereof. *Wishes* and *Electric Dreams* are a series of phrases reminiscent of neon signs, but rather than using gas-filled tubes and electrodes, the (uranium) glass tubing itself glows under UV light.

*Spider Tale* and *What the Birds Knew*, the latter named after Akira Kurosawa’s 1955 film, are both original wire sculptures, with strong ties to environmental injustices surrounding uranium mining. *What the Birds Knew* is a large ant, drawing inspiration from an Aboriginal sacred site in Australia discovered to be near a rich uranium deposit in 1970 (Waggitt, 2001). Folklore held that disturbing the green ants who resided there would lead them to become rampaging monsters.^6^*Spider Tale* is a similarly constructed work, but of a spider. The work is inspired by Japanese and Akan folklore, the former of travelers said to be found caught in spider webs near the “only site in Japan where uranium has been mined,” (artists’ website) and the latter for the Anansi character typically depicted as a spider (Aldred, 2008). Discussion points include:

- The director of the gallery showing *What the Birds Knew* said^7^ that this exhibition expressed “shared cultural expressions of environmental anxieties within Indigenous Australian and Japanese culture, and whether these function as either warnings or premonitions.” Elaborate on this idea and describe how it ties or otherwise relates to previous works considered (e.g., *Nuclear Enchantment*).
- What do you think about *The Crystal Palace* in general? Do you think it impacted you the way the artists intended? Why or why not?

## Findings

### Quantitative Survey Results

**Fig. 5:**
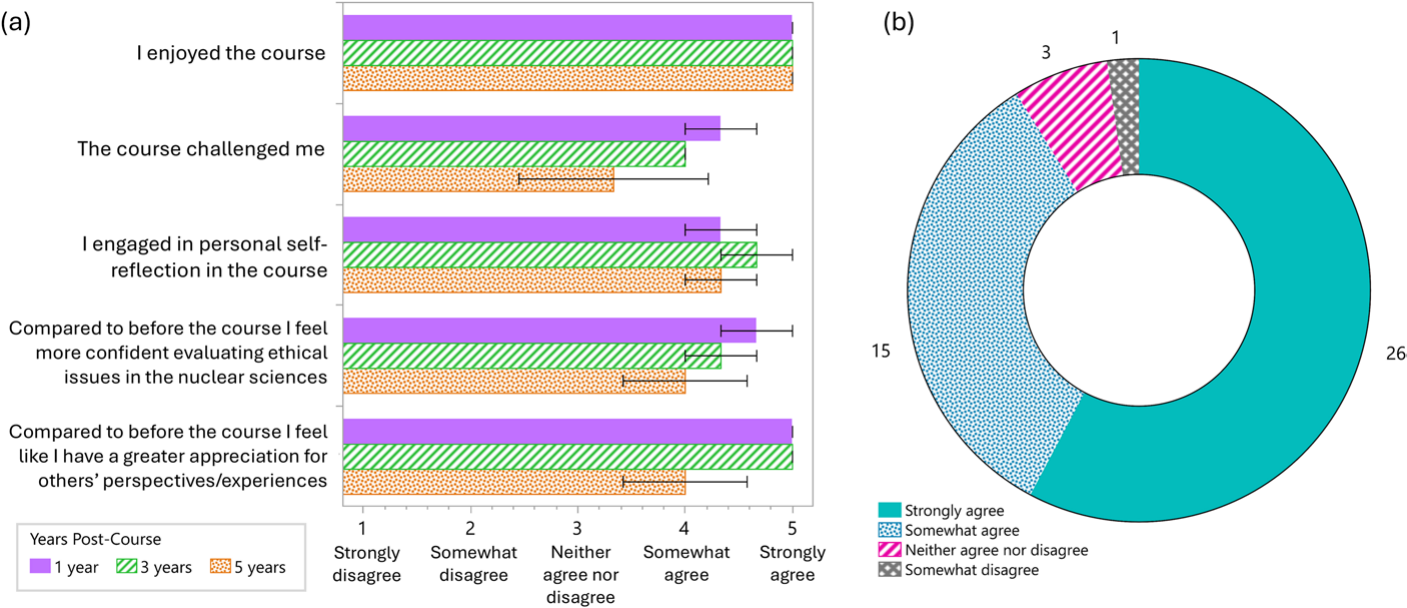
Quantitative results of the student survey. (**a**) Average responses by question and year. Error bars represent standard error of the mean (*n* = 3 each year). (**b**) Total number of responses by response scale; “somewhat agree” or “strongly agree” were suggestive of successful achievement of course goals

Of 13 past students, 69% completed the post-hoc survey (*n* = 9). All students were graduate students during the course, a mix between MS and PhD students. Respondents equally represented cohorts of course participants; one third had completed the course in each 2020, 2022, and 2024 (5, 3, and 1-year post-course, respectively). Quantitative survey results are shown by year in Fig. 5 (mean ± standard error). Students unanimously responded that they enjoyed the course (“strongly agree”). All students agreed (“strongly” or “somewhat”) that they engaged in personal self-reflection. 78% of students agreed that the course challenged them. 89% of students agreed that they gained confidence in evaluating ethical issues in the nuclear sciences, and that they have a greater appreciation for others’ perspectives/experiences post-course. There were no “strongly disagree” responses and only 1 “somewhat disagree” response (“the course challenged me”).

Although the small sample size prevents drawing definitive conclusions, the largely positive responses suggest that the pedagogical approach has been successful in having a positive influence on the student experience and that further research into the integration of art and material culture into nuclear curriculum, and perhaps science and engineering curriculum in higher education more broadly, is warranted.

### Qualitative Survey Results

Open-ended survey questions prompted students to describe what they found to be the most (a) positive and (b) negative aspects of the course, and (c) anything else about the course they would like to share. Prevalent themes included employing novel educational approaches; cultivating student engagement and validation; reflecting on ethics, morals, and empathy; and appreciating the societal impact of nuclear. All the quotes in this section are from students’ survey responses.

Survey responses indicated that the course content was novel compared to other technical classes in nuclear engineering and health physics curricula. Students indicated that they were encouraged “to think about topics that are not typical for an engineering curriculum, such as empathy, community, and risk communication.” The course addressed “darker” parts of nuclear history, confronting the “taboo” and “heavy” aspects students confirmed were not often discussed in technical coursework, highlighting the novelty of an integrated STEAM approach in nuclear.

Student engagement—with the subject matter, the instructor, and fellow students—seems to have facilitated a deeper understanding of the content and introduced students to new ideas and opinions. Discussion-based learning was mentioned, positively, by almost all respondents. Negative comments relating to class discussion referred to the lack of real-time discussion in the online offering of the course during the COVID-19 pandemic. The course gave students a “fresh perspective,” turned “a more critical eye” to aspects of the nuclear field, and helped develop “a rounded view of nuclear culture and radiation protection.” Collectively, this suggests that the pedagogical approach enabled a higher level of thoughtful reflection; that prioritizing engagement through discussion-based learning prompted critical thinking and a deeper level of comprehension, implying the successful integration of science and art (i.e., synergies) as consistent with the work of Danielson et al. (2022) mentioned above.

Multiple survey responses highlighted that students felt encouraged to interrogate and articulate their perspectives. The course structure and facilitation created opportunities for reflection and self-expression, allowing “students to feel heard and seen,” suggesting the arts-informed approach was also successful in student validation. Ethical and moral considerations were brought to the foreground, prompting students to contend not only with the societal impacts of nuclear technologies, but with their own positionality. Students mentioned embracing “the humanizing aspect of the course” and improving their “ability to deal with complex ethical challenges.”

While the survey data may not be representative of all course participants, responses to open-ended survey questions yielded unexpectedly rich data from which perspectives could be inferred. Respondents’ willingness to provide substantive comments—for some, several years after completing the class—indicates the course’s lasting impact on their graduate education. Students described the uniqueness of the course and its value; some recommended the course be offered more broadly (i.e., “to more than just the health physics/nuclear engineering folks”). As one student noted, “it is hard to explain to people who haven’t taken the course why it is so valuable.”

### Instructor Perception

This section describes the instructor’s perception of the course. Each instance of the course referenced was led by the same instructor (NM), who is classically trained in radiological protection and radioecology with professional engagement in related issues in ethics and philosophy.

As currently implemented, the course requires a high level of trust and mutual respect between the instructor and the students as well as among the students to be able to have frank, open, and challenging discussions. Maintaining professionalism and sensitivity to others through difficult conversations and debate additionally requires maturity and emotional intelligence across all participants. Discussions were robust with active participation from all students, attributed to small class size and, in most cases, prior existing relationships between students and the instructor, and perhaps the educational setting (excepting 2020, everyone sat around a table in a conference room rather than individual desks).

The most notable of student artefacts have been the student final projects, or the original creative works. With little exception, projects are thoughtful and personal, from the topic or theme to the medium through which student ideas were expressed. Most students tied their personal experience, emotions, and/or relationships to themes covered in the course, the former of which was not a specific requirement of the assignment. This suggests that students felt a personal connection to the course material, or that the course otherwise inspired self-reflection and awareness. This is consistent with the work of Kinsella and Bidinosti (2016) who, although focused on the health sciences, identified “deepening self-awareness” as an emergent insight (among several) of their arts-informed approach.

With respect to challenges, the course represents a significant time and, if supplying student materials and supplies, monetary investment, as similarly noted by Chatterjee et al. (2015) with respect to object-based learning. Curriculum development necessitates careful review of any works to potentially be considered and curating a selection of materials (drawing from expert curation like that of Carpenter, 2016) to ensure that content is appropriate, coherent, and balanced. Moreover, some elements of the course content are subjective (e.g., whether an individual “likes” something or not, personal impact), but other elements are not (e.g., artist intent and approach, historical and social context). Background research, outside of the instructor’s primary expertise, regarding the difference between the subjective and objective components as well as the depth and meaning of the course materials is challenging but necessary to effectively develop discussions, offer substantive feedback, and lead students in how to perform similar research. Ideally, students are supplied with hard copy or digital versions of the works considered as relevant or possible. Obtaining objects or hands-on activity supplies can involve a meaningful logistical challenge and/or financial cost (Chatterjee et al., 2015) depending on the type or number of activities and number of students, and this cost may extend across course offerings for those items that are consumable. The financial burden can be offset somewhat by utilizing University library services and/or digital copies of certain works or potentially from internal or external grants. Finally, although the instructor’s department currently remains supportive of continuing to offer the course, it is recognized that with many universities implementing minimum class sizes and with emphasis at the graduate level on taking courses for the express purpose of developing research skills directly applicable to thesis or dissertation work, generating enough student (and faculty) interest to garner institutional support over the long term may be challenging.

## Conclusion

Contextualizing the radiological and nuclear sciences in terms of ethics and broader societal impact and perspective through art and nuclear materials culture was intended to help (radiological and nuclear) science and engineering graduate students develop a deeper understanding of social responsibility and empathy as well as work towards “co-construction of peace” (Børsen et al., 2021). Although the course has only been offered a few times so far, it has been well received by students (Fig. 5); notably, students have commented on the usefulness, importance, and uniqueness of the course, in addition to finding it enjoyable. Evaluation, as consideration of student and instructor perceptions, suggests that art in various forms can be incorporated into graduate-level curriculum to improve the educational experience of nuclear-focused students and promote deeper reflection and understanding of social and ethical issues related to their chosen field. This small-scale case study contributes to the call for more robust integration of the arts in STEAM education (Sanz-Camarero et al., 2023), although future work would benefit from more formalized design, robust analysis of student artefacts, and ideally a larger number of student participants.

“Nuclear” STEAM education is likely to become increasingly important as it is well-recognized that technical expertise is necessary, but not sufficient, for effective radiological risk communication and meaningful stakeholder engagement (ICRP, 2018; Till, 2011; Wieder et al., 2022), which many of our students may ultimately pursue as a career. In other words, it is essential for nuclear or radiological protection professionals to be able to engage in decision-making that is rooted in the co-expertise process, which in turn necessitates compassion and understanding, for collaborative governance (ICRP, 2006; Wieder et al., 2022).

Going forward, we intend to continue to offer this course, building upon it by (1) expanding content and scope to be appropriate for other majors and to be openly shared with other universities/schools and (2) developing companion modules for the public to be available via the web. We are also building out a Library Guide (“LibGuide”) with assistance and support from a university librarian to highlight some of the works considered.^8^ Ultimately, we hope to provide open educational resources for a broad spectrum of interested parties that support holistic human capital development.

## Data Availability

Anonymized survey data is available as a supplemental online file.

## Footnotes

1 The warm-up exercise for this discussion is free-writing surrounding a (physical) *akabeko* bobblehead, a red cow folk toy from Fukushima Prefecture, with a subsequent introduction to and discussion of the cultural significance, particularly in the context of recovery and resilience following the triple disaster.

2 A slice of layer cake with a dollop of red, flame-like icing with a round, yellow background and “YELLOW CAKE” written in English across the top

3 See https://www.andrewsmithgallery.com/exhibitions/patricknagatani/estate/nuclear.html Last accessed May 14, 2024.

4
https://gabriellahirst.com/How-To-Make-A-Bomb Last accessed May 14, 2024.

5 Most of the information in this section is drawn from the artists’ website: https://kenandjuliayonetani.com/en/.

6 Reported in TIME Magazine: “AUSTRALIA: Wrath of the Green Ants,” September 9, 1974, https://time.com/archive/6837933/australia-wrath-of-the-green-ants/.

7
http://www.kenandjuliayonetani.com/whatthebirdsknew.pdf.

8
https://clemson.libguides.com/NuclearCulture.

## References

[CR2] Aguilera, D., & Ortiz-Revilla, J. (2021). STEM vs. STEAM education and student creativity: A systematic literature review. *Education Sciences*, *11*(7). 10.3390/educsci11070331

[CR1] Aguilera, D., Lupiáñez, J. L., Vílchez-González, J. M., & Perales-Palacios, F. J. (2021). In search of a long-awaited consensus on disciplinary integration in STEM education. *Mathematics*, *9*(6), 597. https://www.mdpi.com/2227-7390/9/6/597

[CR3] Aldred, B. G. (2008). Anansi. In D. Haase (Ed.), *The Greenwood encyclopedia of folktales and fairy tales* (pp. 30–31). Greenwood. https://archive.org/details/unset0000unse_w7y9/page/30/mode/2up

[CR4] Aleksievich, S., & Gessen, K. (2005). *Voices from Chernobyl* (1st ed.). Dalkey Archive Press. Table of contents http://www.loc.gov/catdir/toc/fy0606/2004063332.html

[CR6] Ando, R. (2018). Trust-what connects science to daily life. *Health Physics*, *115*(5), 581–589. 10.1097/HP.0000000000000945 30260848 10.1097/HP.0000000000000945

[CR5] Andō, H., Trede, M., & Bichler, L. (2010). *Hiroshige: One hundred famous views of Edo*. Taschen.

[CR7] Børsen, T., Serreau, Y., Reifschneider, K., Baier, A., Pinkelman, R., Smetanina, T., & Zandvoort, H. (2021). Initiatives, experiences and best practices for teaching social and ecological responsibility in ethics education for science and engineering students. *European Journal of Engineering Education*, *46*(2), 186–209. 10.1080/03043797.2019.1701632

[CR8] Brame, C. J. (2019). Chapter 4 - Active learning: The student work that builds understanding. In C. J. Brame (Ed.), *Science teaching essentials* (pp. 61–72). Academic Press. 10.1016/B978-0-12-814702-3.00004-4

[CR9] Braun, V.,and, & Clarke, V. (2006). Using thematic analysis in psychology. *Qualitative Research in Psychology*, *3*(2), 77–101. 10.1191/1478088706qp063oa

[CR10] Brougher, K. (2013). Art and nuclear culture. *Bulletin of the Atomic Scientists*, *69*(6), 11–18. 10.1177/0096340213508697

[CR11] Bryant, P. A., Clement, C., Chapple, C. L., Martinez, N., Lips, M., & Dowds, C. (2024). Perspectives of the role of ICRP and the system of protection in meeting the United Nations sustainable development goals. *Journal of Radiological Protection*, *44*(3). 10.1088/1361-6498/ad7bc3 10.1088/1361-6498/ad7bc3 39288788

[CR78] Callard, A. (2009). Q and A: William Wiley. Smithsonian magazine. Available at: https://www.smithsonianmag.com/arts-culture/q-and-a-william-wiley-147461587/

[CR12] Carpenter, E. (2016). *The nuclear culture source book*. Black Dog Publishing.

[CR13] Chatterjee, H. J., Hannan, L., & Thomson, L. (2015). An introduction to object-based learning and multisensory engagement. In *Engaging the senses: Object-based learning in higher education* (pp. 1–20). Ashgate Publishing Ltd.

[CR14] Cojan, M., Colas, F., Fritz, P., Renout, A., Stoica, C., & Ung, M. (2024). Control engineering and mathematics: A new hands-on pedagogical approach through the perspective of art. *IFAC-PapersOnLine*, *58*(3), 88–93. 10.1016/j.ifacol.2024.07.131

[CR15] Costa, A. L., & Kallick, B. (2008). Learning through reflection. In A. L. Costa, & B. Kallick (Eds.), *Learning and leading with habits of mind* (pp. 221–235). ASCD.

[CR16] Cyranoski, D. (2019). The CRISPR-baby scandal: What’s next for human gene-editing. *Nature*, *566*(7745), 440–442. 10.1038/d41586-019-00673-1 30809070 10.1038/d41586-019-00673-1

[CR17] D’Olimio, L., & Peterson, A. (2018). The ethics of narrative art: Philosphy in schools, compassion and learning from stories. *Journal of Philosophy in Schools*, *5*(1), 92–110. https://www.ojs.unisa.edu.au/index.php/jps/article/view/1487

[CR18] Danielson, R. W., Grace, E., White, A. J., Kelton, M. L., Owen, J. P., Fisher, S., Diaz Martinez, K., A., & Mozo, M. (2022). Facilitating systems thinking through arts-based STEM integration [Original Research]. *Frontiers in Education*, *7*. 10.3389/feduc.2022.915333 10.3389/feduc.2022.915333 PMC10817773 38283981

[CR19] Dant, T. (2015). Material culture. In *The Blackwell Encyclopedia of Sociology*. 10.1002/9781405165518.wbeosm046.pub2

[CR20] Decamous, G. (2018). *Invisible colors: The arts of the atomic age*. The MIT Press.

[CR21] Dedoose. (2025). *Version 10.0.25, cloud application for managing, analyzing, and presenting qualitative and mixed method research data*. SocioCultural Research Consultants. LLC. https://www.dedoose.com/

[CR22] Delany, C., & Gaunt, H. (2018). I left the museum somewhat changed: Visual arts and health ethics education. *Cambridge Quarterly of Healthcare Ethics*, *27*(3), 511–524. 10.1017/S0963180117000913 29845924 10.1017/S0963180117000913

[CR23] Dyment, J. E., & O’Connell, T. S. (2010). The quality of reflection in student journals: A review of limiting and enabling factors. *Innovative Higher Education*, *35*(4), 233–244. 10.1007/s10755-010-9143-y

[CR24] Grauer, K. (2012). A case for case study research in education. In S. R. Klein (Ed.), *Action research methods: Plain and simple* (pp. 69–79). Palgrave Macmillan US. 10.1057/9781137046635_4

[CR25] Hamilton, L., & Corbett-Whittier, C. (2013). *Using case study in education research*. SAGE Publications Ltd. 10.4135/9781473913851

[CR26] Hartikainen, S., Rintala, H., Pylväs, L., & Nokelainen, P. (2019). The concept of active learning and the measurement of learning outcomes: A review of research in engineering higher education. *Education Sciences*, *9*(4), 276. https://www.mdpi.com/2227-7102/9/4/276

[CR27] Hirst, G., & Harper, W. (2019). *How to make a bomb* (1st ed.). The Old Waterworks.

[CR28] Hirst, G., & Harper, W. (2021). *An english garden*. The Old Waterworks.

[CR29] Howell, R. A. (2021). Engaging students in education for sustainable development: The benefits of active learning, reflective practices and flipped classroom pedagogies. *Journal of Cleaner Production*, *325*. 10.1016/j.jclepro.2021.129318

[CR30] Hughes, J. (2012). What is British nuclear culture? Understanding uranium 235. *British Journal for the History of Science*, *45*(166), 495–518. 10.1017/S0007087412001021

[CR31] ICRP. (2006). The optimisation of radiological protection: Broadening the process. ICRP publication 101b. *Annals of the Icrp*, *36*(3), 65, 71–104. 10.1016/j.icrp.2006.09.007 10.1016/j.icrp.2006.09.007 17174715

[CR32] ICRP. (2007). The 2007 recommendations of the international commission on radiological protection. ICRP publication 103. *Annals of the Icrp*, *37*(2–4), 1–332. 10.1016/j.icrp.2007.10.003 10.1016/j.icrp.2007.10.003 18082557

[CR33] ICRP. (2018). Ethical foundations of the system of radiological protection. ICRP publication 138. *Annals of the ICRP *, *47*(1), 1–65. 10.1177/0146645317746010 10.1177/0146645317746010 29457463

[CR34] ICRP. (2020). Radiological protection of people and the environment in the event of a large nuclear accident: Update of ICRP publications 109 and 111. ICRP publication 146. *Annals of the ICRP*, *49*(4), 11–135. 10.1177/0146645320952659 10.1177/0146645320952659 33291942

[CR35] Jolivette, C. (2012). Science, art and landscape in the nuclear age. *Art History*, *35*(2), 252–269. 10.1111/j.1467-8365.2011.00885.x

[CR36] Kiger, M. E.,and, & Varpio, L. (2020). Thematic analysis of qualitative data: AMEE guide 131. *Medical Teacher*, *42*(8), 846–854. 10.1080/0142159X.2020.1755030 32356468 10.1080/0142159X.2020.1755030

[CR37] Kinsella, E. A., & Bidinosti, S. (2016). I now have a visual image in my mind and it is something I will never forget’: An analysis of an arts-informed approach to health professions ethics education. *Advances in Health Sciences Education*, *21*(2), 303–322. 10.1007/s10459-015-9628-7 26245943 10.1007/s10459-015-9628-7

[CR38] Kreth, Q., Schiff, D. S., Lee, J., Borenstein, J., & Zegura, E. (2024). Social responsibility and ethics in STEM education: The state of the field. In E. Hildt, K. Laas, E. M. Brey, & C. Z. Miller (Eds.), *Building inclusive ethical cultures in STEM* (pp. 19–33). Springer . 10.1007/978-3-031-51560-6_2

[CR39] Kuo, H. C. (2024). Transforming tomorrow: A practical synthesis of STEAM and PBL for empowering students’ creative thinking. *International Journal of Science and Mathematics Education*. 10.1007/s10763-024-10511-0

[CR40] Law, J. M. (1994). Violence, ritual reenactment, and ideology: The Hōjō-e (Rite for release of sentient Beings) of the USA Hachiman shrine in Japan. *History of Religions*, *33*(4), 325–357. http://www.jstor.org/stable/1062714

[CR41] Malone, J. (2013). Schrodinger: Risking mystery and creativity in science. *ARTS (Arts in Religious and Theological Studies)*, *25*, 27–39.

[CR42] Malone, J. (2022). Reflections and images: A place for art in medical physics? *Physica Medica*, *98*, 63–79. 10.1016/j.ejmp.2022.04.004 35500305 10.1016/j.ejmp.2022.04.004

[CR43] Marcone, G. (2022). Humanities and social sciences in relation to sustainable development goals and STEM education. *Sustainability*, *14*(6), 3279. https://www.mdpi.com/2071-1050/14/6/3279

[CR44] Martinez, N. E. (2020). The 2018 Bo Lindell laureate lecture: Finding common ground between science, ethics, and experience. *Annals of the ICRP*, *49*(1_suppl), 9–31. 10.1177/0146645320946618 33047613 10.1177/0146645320946618

[CR45] Martinez, N. E., Jokisch, D. W., Dauer, L. T., Eckerman, K. F., Goans, R. E., Brockman, J. D., Tolmachev, S. Y., Avtandilashvili, M., Mumma, M. T., Boice, J. D. Jr., & Leggett, R. W. (2022). Radium dial workers: Back to the future. *International Journal of Radiation Biology*, *98*(4), 750–768. 10.1080/09553002.2021.1917785 33900890 10.1080/09553002.2021.1917785 PMC10563809

[CR46] Matthew, B. J., Homero, M., Jason, F., Lilianny, V., & Pamela, L. D. (2020). *Exploring perceptions of disciplines using arts-informed methods* Virtual On line. https://peer.asee.org/34644

[CR47] Mejias, S., Thompson, N., Sedas, R. M., Rosin, M., Soep, E., Peppler, K., Roche, J., Wong, J., Hurley, M., Bell, P., & Bevan, B. (2021). The trouble with STEAM and why we use it anyway. *Science Education*, *105*(2), 209–231. 10.1002/sce.21605

[CR48] Merriam, S. B. (1985). The case study in educational research: A review of selected literature. *The Journal of Educational Thought (JET) / Revue de La Pensée Éducative*, *19*(3), 204–217. http://www.jstor.org/stable/23768608

[CR49] Michael, J. (2006). Where’s the evidence that active learning works? *Advances in Physiology Education*, *30*(4), 159–167. 10.1152/advan.00053.2006 17108243 10.1152/advan.00053.2006

[CR50] Nagatani, P., & Parry, E. (1991). *Nuclear enchantment* (1st edn.). University of New Mexico.

[CR51] Oughton, D. (2013). Ethical aspects of ecological risks from radiation. *Social and Ethical Aspects of Radiation Risk Management*, *19*, 71–85. 10.1016/b978-0-08-045015-5.00005-8

[CR52] Park, M., Leahey, E., & Funk, R. J. (2023). Papers and patents are becoming less disruptive over time. *Nature*, *613*(7942), 138–144. 10.1038/s41586-022-05543-x 36600070 10.1038/s41586-022-05543-x

[CR53] Perales, F. J.,and, & Aróstegui, J. L. (2024). The STEAM approach: Implementation and educational, social and economic consequences. *Arts Education Policy Review*, *125*(2), 59–67. 10.1080/10632913.2021.1974997

[CR54] Perko, T., Van Oudheusden, M., Turcanu, C., Polzl-Viol, C., Oughton, D., Schieber, C., Schneider, T., Zolzer, F., Mays, C., Martell, M., Baude, S., de Witte, C., Prlic, I., Cantone, I., Salomaa, M. C., Duranova, S., Economides, T., S., & Molyneux-Hodgson, S. (2019). Towards a strategic research agenda for social sciences and humanities in radiological protection. *Journal of Radiological Protection*, *39*(3), 766–784. 10.1088/1361-6498/ab0f89 30865935 10.1088/1361-6498/ab0f89

[CR55] Rentetzi, M., & Ito, K. (2021). The material culture and politics of artifacts in nuclear diplomacy. *Centaurus*, *63*(2), 233–243. 10.1111/1600-0498.12394

[CR76] Rigby, A. (1998). A peace symbol’s origins. *Peace Review, 10*(3), 475–479. 10.1080/10402659808426187

[CR56] Roberts, S. (2017). Patrick Nagatani, Photographer famous for collages, dies at 72. *The New York Times*. https://www.nytimes.com/2017/11/13/obituaries/patrick-nagatani-photographer-famous-for-collages-dies-at-72.html

[CR57] Ross, L. M. (2024). Nuclear cultural heritage: From energy past to heritage future. *Heritage and Society*, *17*(2), 296–315. 10.1080/2159032x.2023.2266644

[CR58] Rowland, R. E. (1994). *Radium in humans: A review of U.S. Studies*. Argonne National Laboratory.

[CR59] Ryan, M. (2013). The pedagogical balancing act: Teaching reflection in higher education. *Teaching in Higher Education*, *18*(2), 144–155. 10.1080/13562517.2012.694104

[CR60] Sanz-Camarero, R., Ortiz-Revilla, J., & Greca, I. M. (2023). The impact of integrated STEAM education on arts education: A systematic review. *Education Sciences*, *13*(11), 1139. https://www.mdpi.com/2227-7102/13/11/1139

[CR61] Smith, G., & Martinez, N. (2017). Ethics, stakeholders and low doses. *Journal of Radiological Protection*, *37*(4), 947–952. 10.1088/1361-6498/aa9600 29068323 10.1088/1361-6498/aa9600

[CR62] The Old Waterworks (2023, September 12, 2021). *How to make a bomb workshop at FPG*. https://vimeo.com/792248978

[CR63] Till, J. E. (2011). Building trust, credibility, and respect in environmental risk assessment. *Health Physics News*, *39*(5).

[CR64] Trede, M., Andō, H., & Bichler, L. & Ukiyoe Ōta Kinen bijutsukan. (2015). *Hiroshige: Meisho Edo hyakkei = one hundred famous views of Edo* [still image]. Taschen.

[CR65] Trout, I. Y., Tose, S., Caswell, C., & Christensen, M. C. (2022). Integrating arts in a collaborative research process: An arts-Informed inquiry. *Learning Landscapes*, *15*(1), 367–384.

[CR66] Verma, A. (2021). The nuclear, humanities, and social science nexus: Challenges and opportunities for speaking across the disciplinary divides. *Nuclear Technology*, *207*(9), iii–xv. 10.1080/00295450.2021.1941663

[CR67] Volkmar, A. (2022). *Art and nuclear power: The role of culture in The environmental debate*. Lexington Books.

[CR68] Waggitt, P. W. (2001). The decommissioning and rehabilitation of the Nabarlek Uranium Mine, northern Australia. Workshop on the rehabilitation of Nabarlek Uranium Mine. 18–19 April 2000. Supervising Scientist Report 160, Darwin NT, Australia.

[CR69] Wieder, J. S., Schneider, T., & Martinez, N. E. (2022). The three R’s of reasonable in radiological protection: Relationships, rationale, and resources. *Journal of Radiological Protection*, *42*(2). 10.1088/1361-6498/ac563b 10.1088/1361-6498/ac563b 35176730

[CR70] Wong, J. T., Bui, N. N., Fields, D. T., & Hughes, B. S. (2023). A learning experience design approach to online professional development for teaching science through the arts: Evaluation of teacher content knowledge, self-efficacy and STEAM perceptions. *Journal of Science Teacher Education*, *34*(6), 593–623. 10.1080/1046560X.2022.2112552

[CR71] Yanai, I., & Lercher, M. J. (2023). Make science disruptive again. *Nature Biotechnology*, *41*(4), 450–451. 10.1038/s41587-023-01736-5 10.1038/s41587-023-01736-5 36973558

[CR72] Yin, R. K. (2018). *Case study research and applications: Design and methods* (Sixth ed.). SAGE.

[CR73] Zandvoort, H., Børsen, T., Deneke, M., & BirdS. J. (2013). Editors’ overview perspectives on teaching social responsibility to students in science and engineering. *Science and Engineering Ethics*, *19*(4), 1413–1438. 10.1007/s11948-013-9495-7 24277690 10.1007/s11948-013-9495-7

[CR74] Zhang, C., & Jia, B. (2024). Enriching STEAM education with visual art: Education benefits, teaching examples, and trends. *Discover Education*, *3*(1), 247. 10.1007/s44217-024-00354-w

[CR75] Zölzer, F., & Zölzer, N. (2022). The role of empathy in ethics of radiological protection. *Journal of Radiological Protection*, *42*(1), 014002. 10.1088/1361-6498/ac3ccb 10.1088/1361-6498/ac3ccb 34818639

